# Microtrabecular Fracture with Intramedullary Fat Globules

**DOI:** 10.5334/jbsr.3317

**Published:** 2023-11-02

**Authors:** Jeroen Bauwens, Koenraad Nieboer

**Affiliations:** 1 UZ Brussel, BE; 2 UZ Jette, BE

**Keywords:** microtrabecular fracture, intramedullary fat globules, bone marrow edema, fat coalescence, dual-energy CT, MRI

## Abstract

**Teaching Point:** In rare cases, trauma may result in intramedullary fat globules which have a characteristic aspect both on MRI and dual-energy CT.

## Case History

A 29-year-old man came to the emergency care unit after injuring his hand. He had mild swelling on the ulnar side of his right hand, as well as pain at the level of metacarpal IV during active movement. An X-ray of the hand showed no bony lesion, and the patient was discharged.

**Figure 1:**
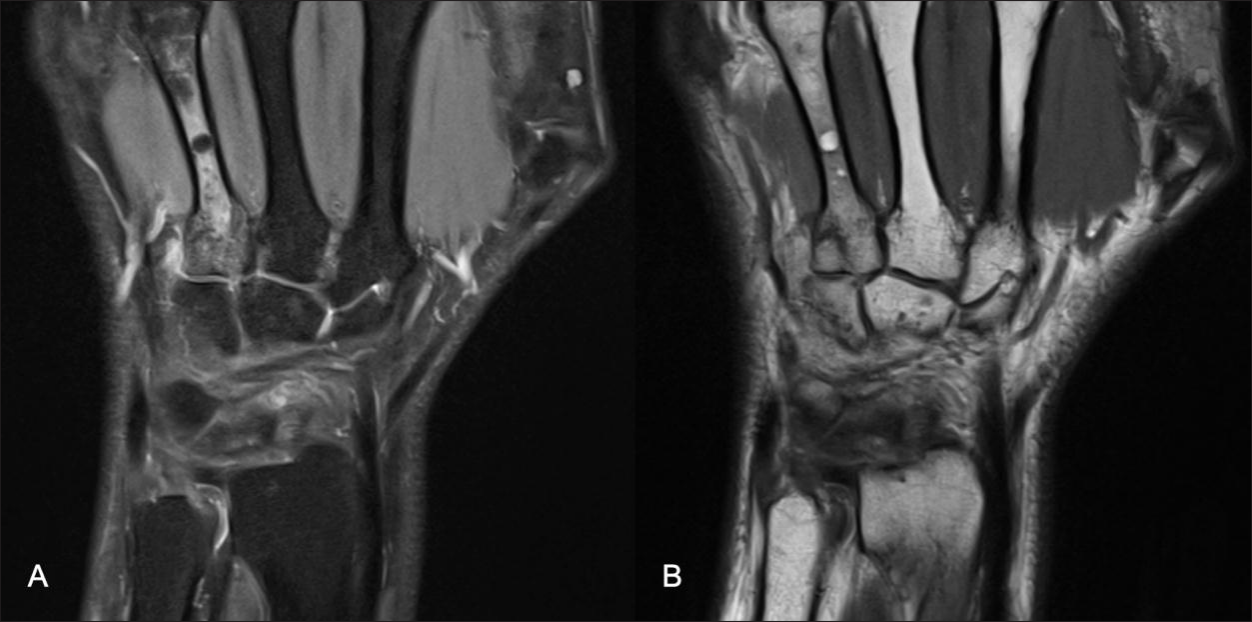


One month later the patient received an MRI scan of the wrist due to persistent pain on the ulnar side. A microtrabecular fracture was revealed at the base of metacarpal IV (Figure 1B, PD coronal plane). A large amount of edema could be seen in the medulla of the metacarpal bone (Figure 1A, PD fat sat, coronal plane). Surprisingly, there were also small globules of fat (4 mm) embedded in the diaphysis.

**Figure 2:**
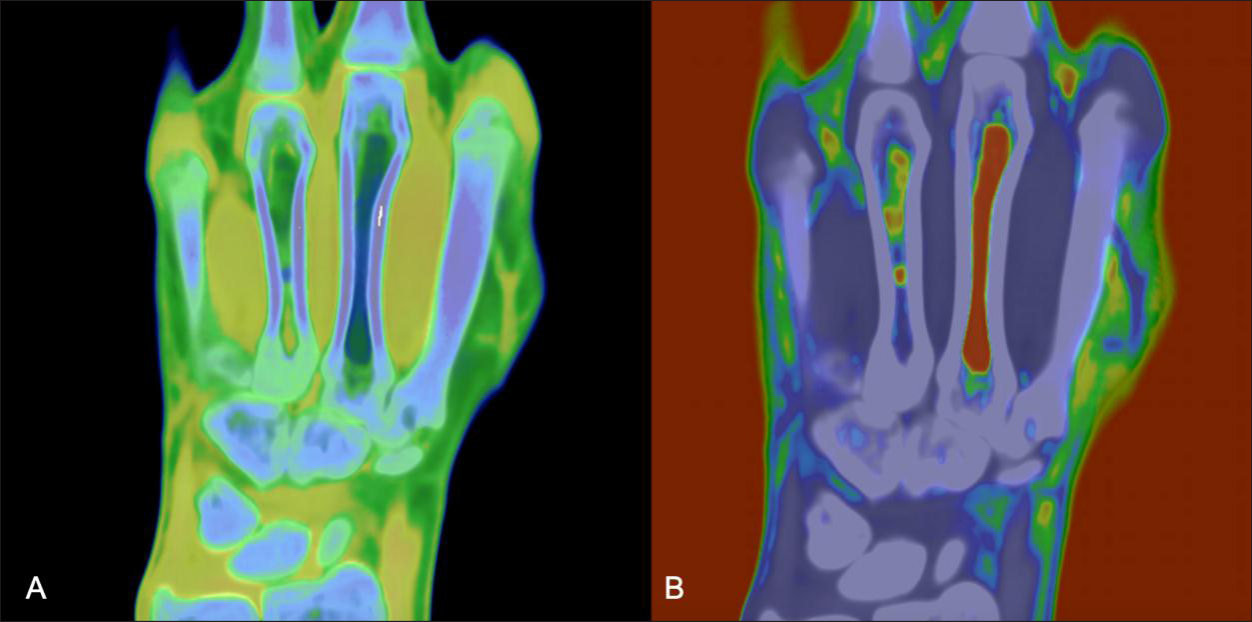


A dual-energy computed tomograpy (DECT) scan was performed to confirm the diagnosis. No fracture line was seen. Water reconstructions of the patient’s right hand showed massive bone marrow edema in metacarpal IV (Figure 2A). Fat reconstructions confirmed small fat globules at the diaphyseal level and an associated relative absence of fatty yellow marrow elsewhere in the bone (Figure 2B).

## Comments

In medical literature, manifestations of extraosseous fat accumulation following trauma have been well-documented, including systemic fat embolism, lipohemarthrosis, fat around tendon sheaths and subperiosteal fat accumulation. Intramedullary fat globules are a relatively infrequent finding though, believed to occur in no more than 1.9% of bone contusion or fracture [1].

With osseous injury, cell death and disrupted blood supply lead to hemorrhage and inflammation in fatty marrow, resulting in necrosis of fatty marrow cells in the region. As a consequence, liquefied fat is released and traverses through interconnected trabecular chambers to sites distant from the injury, with some observed up to 6 cm away from the fracture site. This phenomenon aligns with the mechanical theory of fat embolism in the context of fractures, where increased intramedullary pressure due to hemorrhage and inflammatory edema may force fat globules into torn veins [1].

Intramedullary fat globules are more commonly seen in older patients (although our patient is quite young). This is likely due to age-related bone loss and alterations in trabecular architecture facilitating the migration of liquefied fat.

Our case demonstrates the peculiar aspect of intramedullary fat globules after trauma, even without a clear fracture line on X-ray and CT. Our case also highlights the usefulness of DECT in confirming certain pathophysiologies on a microscopic level. Fat reconstructions clearly showed a decrease of fatty bone marrow due to liquefaction and coalescence secondary to trauma.
